# From Infertility to Motherhood: Psychological Experiences of Women in the Perinatal Period After Fertility Treatment

**DOI:** 10.1192/j.eurpsy.2026.12036

**Published:** 2026-07-31

**Authors:** J. P. C. M. Silva, C. Hinds, N. Nguyen, S. Coyle

**Affiliations:** 1 Rotunda Hospital; 2 National Maternity Hospital; 3 St James`Hospital; 4 The Coombe Hospital, Dublin, Ireland

## Abstract

**Introduction:**

Infertility and its treatments have profound psychological consequences, yet little is known about how these experiences shape women’s journeys through pregnancy, birth, and early motherhood. Existing research shows high levels of distress during assisted reproductive treatment, but less attention has been paid to the persistence of this vulnerability across the perinatal period.

**Objectives:**

This study aimed to explore the lived experiences of mothers who conceived through fertility treatment in Ireland, focusing on how infertility and assisted reproductive technologies (ART) influenced their mental health and sense of identity during pregnancy, delivery, and the postnatal period.

**Methods:**

A qualitative study design was used. Fifteen mothers were recruited from two Specialist Perinatal Mental Health Services. Semi-structured interviews were conducted remotely via Zoom, lasting 60–90 minutes. Interviews explored participants’ fertility journey, pregnancy, birth experiences, and transition to motherhood. Transcripts were analysed using Interpretative Phenomenological Analysis (IPA), supported by NVivo software, to ensure participants’ perspectives remained central.

**Results:**

Analysis of nine complete interviews identified five themes across a temporal trajectory: (1) the shock of infertility diagnosis and its disruption of identity; (2) navigating fertility treatment, described as invasive, exhausting, and isolating; (3) pregnancy after ART, often marked by anxiety, hypervigilance, and fear of loss; (4) birth and delivery, where operative interventions such as caesarean section were sometimes felt as further evidence of bodily failure; and (5) early motherhood, characterised by joy but also delayed bonding, breastfeeding challenges, exhaustion, and silencing due to expectations of gratitude. Across stages, women reported limited access to psychological support and negative encounters with healthcare staff.

**Conclusions:**

The psychological burden of infertility does not resolve with conception. Instead, infertility identity, treatment trauma, and postnatal challenges intertwine to create a continuum of vulnerability. These findings underscore the need for fertility and maternity services to embed psychological support into routine care, extend mental health screening across the perinatal timeline, and adopt trauma-informed, gender-sensitive practices to meet the needs of women following fertility treatment.

**Disclosure of Interest:**

None Declared

